# CAF-1 promotes efficient PrimPol recruitment to nascent DNA for single-stranded DNA gap formation

**DOI:** 10.1093/nar/gkae1068

**Published:** 2024-11-18

**Authors:** Joshua Straka, Jude B Khatib, Lindsey Pale, Claudia M Nicolae, George-Lucian Moldovan

**Affiliations:** Department of Biochemistry and Molecular Biology, The Pennsylvania State University College of Medicine, Hershey, PA 17033, USA; Department of Biochemistry and Molecular Biology, The Pennsylvania State University College of Medicine, Hershey, PA 17033, USA; Department of Biochemistry and Molecular Biology, The Pennsylvania State University College of Medicine, Hershey, PA 17033, USA; Department of Biochemistry and Molecular Biology, The Pennsylvania State University College of Medicine, Hershey, PA 17033, USA; Department of Biochemistry and Molecular Biology, The Pennsylvania State University College of Medicine, Hershey, PA 17033, USA

## Abstract

Suppression of single-stranded DNA (ssDNA) gap accumulation at replication forks has emerged as a potential determinant of chemosensitivity in homologous recombination (HR)-deficient tumors, as ssDNA gaps are transformed into cytotoxic double-stranded DNA breaks. We have previously shown that the histone chaperone CAF-1’s nucleosome deposition function is vital to preventing degradation of stalled replication forks correlating with HR-deficient cells’ response to genotoxic drugs. Here we report that the CAF-1–ASF1 pathway promotes ssDNA gap accumulation at replication forks in both wild-type and breast cancer (BRCA)-deficient backgrounds. We show that this is independent of CAF-1’s nucleosome deposition function but instead may rely on its proper localization to replication forks. Moreover, we show that the efficient localization to nascent DNA of PrimPol, the enzyme responsible for repriming upon replication stress, is dependent on CAF-1. As PrimPol has been shown to be responsible for generating ssDNA gaps as a byproduct of its repriming function, CAF-1’s role in its recruitment could directly impact ssDNA gap formation. We also show that chemoresistance observed in HR-deficient cells when CAF-1 or ASF1A are lost correlates with suppression of ssDNA gaps rather than protection of stalled replication forks. Overall, this work identifies an unexpected role of CAF-1 in regulating PrimPol recruitment and ssDNA gap generation.

## Graphical Abstract

**Graphical Abstract:**
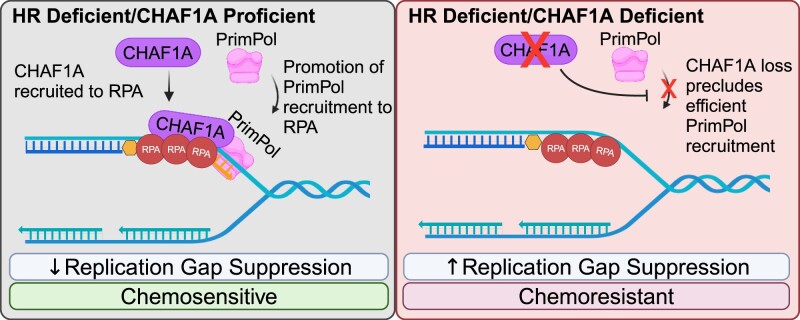

## Introduction

Though major advances in mechanistic understanding of cancers have improved patient prognoses, treatment with conventional chemotherapeutics which drive DNA damage are still widely used. The exact mechanism through which genotoxic therapies such as the platinum compound cisplatin and Poly(ADP-ribose) Polymerase (PARP) inhibitors (PARPis) induce sensitivity in cancer cells is still not well understood and prediction of individual patient responses and what drives them remain unclear. Breast cancer susceptibility genes (BRCA) deficient cancers are common candidates for use of these genotoxic treatments. BRCA1 and BRCA2 act as tumor suppressors by ensuring that repair of double strand breaks (DSBs) can occur using HR, an error-free mechanism of DSB repair. BRCA1 and BRCA2 also share roles in maintaining genomic integrity through protecting reversed replication forks, known as fork protection (FP) (1), as well as suppressing the accumulation of single-stranded DNA (ssDNA) gaps during DNA replication, known as replication gap suppression (RGS) (2–6). Maintenance of reversed replication forks is carried out through BRCA1/2-mediated stabilization of RAD51 filaments on reversed arms of replication forks under replication stress (1), preventing nucleolytic degradation of the reversed arm by MRE11 and EXO1 (7). In this way, the BRCA proteins work to prevent DNA damage rather than to repair it. Suppression of replication-associated ssDNA gap accumulation by the BRCA proteins is achieved through two separate mechanisms: restraint of replication forks preventing PrimPol-mediated repriming under replication stress (3–6) and promotion of post-replicative repair of ssDNA gaps (2,8,9). PrimPol is the enzyme responsible for carrying out repriming and restart of stalled DNA synthesis in human cells (10–12). PrimPol is a member of the Archaeo-Eukaryotic Primase (AEP) superfamily of primases (13) and has both primase and polymerase activities. Importantly, as a consequence of its repriming function, PrimPol leaves behind ssDNA gaps on nascent DNA as it begins repriming downstream of the location of the lesion that previously stalled synthesis (14). Thus, PrimPol function in repriming DNA synthesis represents an important source of ssDNA gap generation. Interestingly, FP and RGS are two mechanisms that have been postulated to inform drug response outside of the context of HR deficiency (4,15,16). Recent publications have demonstrated that RGS may correlate more closely with the response to genotoxic therapy than does restoration of FP (4,16). On the other hand, the investigation of BRCA2 separation of function mutants proficient in HR but deficient in FP and RGS suggests that the ultimate determinant of chemosensitivity is HR (17).

The Chromatin Assembly Factor 1 (CAF-1) complex is a major effector in the pathway responsible for both de-novo establishment and recycling of nucleosomes at actively replicating DNA. CAF-1 is a multi-unit complex made up of three subunits: CHAF1A, CHAF1B and p48. The complex is targeted to replicating DNA by CHAF1A interaction with proliferating cell nuclear antigen (PCNA), the sliding clamp-like structure which provides processivity to DNA polymerases Polϵ and Polδ and serves as a scaffold for recruitment of replication factors (18–20), through a PCNA-interacting peptide (PIP) motif. Mediated by its CHAF1B subunit, CAF-1 cooperates with a second histone chaperone, namely Anti-Silencing Factor 1 (ASF1), which is responsible for shuttling H3-H4 dimers from the cytosol to the nucleus and delivering them to CAF-1 (21). During replication, CAF-1 initiates the first step of nucleosome establishment by depositing H3-H4 heterodimers on DNA. New H2A and H2B dimers are then delivered to DNA via additional histone chaperones, either NAP-1 or FACT, to complete nucleosome assembly.

Along with its role in DNA replication in S-phase, CAF-1 also has roles in DNA repair. Historically, CAF-1 has been implicated in a DNA repair-linked chromatin establishment pathway that is triggered upon nucleotide excision repair (NER) of ssDNA gaps and breaks (22,23). This process is an ATP-dependent process that requires PCNA interaction and results in re-establishment of nucleosomes at sites of repair (22,23). CHAF1A also is recruited to DSB sites through a direct interaction with the KU70/KU80 complex and the protein 14–3-3 ζ. It was hypothesized that CAF-1 is recruited independently of PCNA to these sites because as opposed to NER, there is no need for long range DNA synthesis while the need for nucleosome assembly persists (24). More recently, we identified a role for CAF-1 in maintaining FP (9,25). We found that CAF-1-mediated protection of reversed replication forks does not operate correctly in HR deficient cells due to a defect in Okazaki fragment maturation, thus rendering them susceptible to genotoxic agents through degradation of reversed replication forks. Restoration of nucleosome assembly in this context can represent a potential resistance mechanism of HR-deficient cells to therapy (25).

Here, we demonstrate that the histone chaperones CAF-1 and ASF1 both control ssDNA gap accumulation and the response to genotoxic drug treatment. Mechanistically, we show that CAF-1 and ASF1 promote ssDNA gap accumulation through enhancing PrimPol recruitment to nascent DNA during replication stress in HR-deficient backgrounds. Moreover, we show that loss of ASF1A, one of the two ASF1 paralogs, in BRCA-deficient cells results in cisplatin resistance correlated with RGS rather than FP. Our work identifies an unexpected role for CAF-1 and ASF1 in ensuring efficient PrimPol function and driving chemosensitivity through ssDNA gap generation.

## Materials and methods

### Cell culture and protein techniques

Human 293T, HeLa, RPE1 and MDA-MB-436 cells were grown in Dulbeccos’s modified Eagle’s media (DMEM) supplemented with 10% FBS, and 1% penicillin/streptomycin. 293T, HeLa and MDA-MB-436 cells were obtained from ATCC. CHAF1A-knockout HeLa and 293T cells as well as BRCA2-knockout HeLa cells and BRCA2-knockout PrimPol-overexpressing HeLa cells were created in our laboratory and were previously described (25–27). RPE1-p53^KO^ and RPE1-p53^KO^-BRCA1^KO^ cells were obtained from Dr Alan D’Andrea (Dana-Farber Cancer Institute, Boston, MA).

Small interfering RNA (siRNA)-mediated gene knockdown was performed using Lipofectamine RNAiMAX. The AllStars Negative Control siRNA (Qiagen 1 027 281) was used as a control. The following oligonucleotide sequences (Stealth or SilencerSelect siRNA, ThermoFisher) were used:

CHK1: ID: 5504;

FEN1: ID: S5104;

RPA3: ID: s12133;

RPA2: HSS109321;

DNA2: AAGGAUACAGUUGCCUGCAUUCUAA;

CHAF1A#1: ID: s19499;

CHAF1A#2: ID: s19501;

ASF1A#1: CAGAGAGCAGUAAUCCAAAUCUACA;

ASF1A#2: ID: s226043;

BRCA1: AAUGAGUCCAGUUUCGUUGCCUCUG;

BRCA2: AUUAGGAGAAGACAUCAGAAGCUUG;

PrimPol: ID: 39536.

Whole cell extracts were prepared through denaturation by boiling cells in 100 mM Tris, 4% sodiumdodecyl sulfate (SDS), 0.5 M β-mercaptoethanol. Antibodies used for western blot at 1:500 (unless otherwise noted) were:

CHAF1A (Cell Signaling Technology 5480s);

CHK1 (Cell Signaling Technology 2360s);

FEN1 (Santa Cruz Biotechnology sc-28355);

RPA2 (Abcam AB2175);

RPA3 (Santa Cruz Biotechnology sc-271564);

DNA2 (Abcam AB96488);

ASF1A (Santa Cruz Biotechnology sc-53171);

BRCA1 (Santa Cruz Biotechnology sc-6954);

BRCA2 (Calbiochem OP95);

GAPDH (Santa Cruz Biotechnology sc-47724);

PrimPol (Proteintech 29824–1-AP) (1:250);

Vinculin (Santa Cruz Biotechnology sc-25336).

Chemical compounds used were: mirin (Selleck Chemicals S8096), C5 (28).

### CRISPR screen

The CRISPR knockout screen was performed using the Brunello Human CRISPR Knockout Library (Addgene 73 179) encompassing 76 411 guide RNAs (gRNAs) targeting 19 114 genes. To maintain 250-fold coverage of the library, 55 million cells from each of the 293T WT and 293T CHAF1A^KO^ cell lines were infected at a multiplicity of infection (MOI) of 0.4. Cells were selected for 3 days with 1 μg/ml puromycin. Twenty million library infected cells were plated and 24 h later were either untreated or exposed to cisplatin at a concentration of 0.625 μM for 3 days and then collected. Treatment of the CHAF1A^KO^ 293T cells resulted in 95% viability compared to untreated, while treatment of the 293T wild-type (WT) cells resulted in a 93% viability compared to untreated. Genomic DNA was isolated with the DNeasy Blood and Tissue Kit (Qiagen 69 504) and utilized for polymerase chain reaction (PCR) using Illumina adapters to identify the gRNA representation in each sample. Approximately 10 μg of gDNA was used in each PCR reaction along with 20 μl 5X HiFi Reaction Buffer, 4 μl of P5 primer, 4 μl of P7 primer, 3 μl of Radiant HiFi Ultra Polymerase (Stellar Scientific) and water. The P5 and P7 primers were determined using the user guide provided with the CRISPR libraries (https://media.addgene.org/cms/filer_public/61/16/611619f4-0926-4a07-b5c7-e286a8ecf7f5/broadgpp-sequencing-protocol.pdf). The PCR cycled as follows: 98°C for 2 min before cycling, then 98°C for 10 s, 60°C for 15 s and 72°C for 45 s, for 30 cycles, and finally 72°C for 5 min. The purified PCR product was then sequenced with Illumina HiSeq 2500 single read for 50 cycles, targeting 10 million reads. Sequencing results were analyzed using the MAGeCK algorithm, which compares raw gRNA read counts to test if individual guide abundance varies significantly between the conditions. The CRISPR screen was conducted twice independently, and results are representative of combined MAGeCK analysis in a paired fashion. KEGG and Gene Ontology Pathway enrichment analysis was performed using DAVID.

### Viability/survival assays

For cellular viability assays, 500 siRNA-treated cells were seeded per well in 96-well plates in triplicate either in the presence or absence of drug and incubated for 3 days as indicated. A luminescent ATP-based assay was performed using the CellTiterGlo reagent (Promega G7572) according to the manufacturer’s instructions. Luminescence was quantified using a Promega GloMax Navigator plate reader. For clonogenic survival assays, 1000 siRNA-treated cells were seeded in 6-well plates and treated with drug as indicated. Media was changed after 3 days. Colonies were stained after 10–14 days. Colonies were washed with PBS, fixed with a solution of 10% methanol and 10% acetic acid, and stained with 1% crystal violet (Aqua Solutions).

### Functional assays

Neutral and BrdU alkaline comet assays were performed as previously described using the Comet Assay Kit (Trevigen, 4250–050). For the BrdU alkaline comet assay, cells were incubated with 100 μM BrdU for 30 min along with the addition of chemical compounds (hydroxyurea [HU], cisplatin and mirin), according to the labeling schemes presented. BrdU alkaline comet assays with Olaparib treatment included a 1-h pretreatment with the drug before BrdU incubation. Slides were stained with anti-BrdU (BD347580) and secondary AF568-conjugated antibodies (Invitrogen A-11031). Slides were imaged on a Nikon Eclipse Ts2 microscope operating the NIS Elements V1.10.00 software. Olive tail moment was calculated using CometScore 2.0. The Olive tail moment measurement incorporates information about both the length of comet tails and the distribution of DNA throughout the comet by multiplying comet tail length (distance between the head and end of tail/head diameter) by the percent of DNA contained in the tail, allowing for greater information to be obtained on the distribution of labeled DNA (29,30). Immunofluorescence was performed as previously described (31) using the following primary antibodies: γH2AX antibody (MilliporeSigma JBW301) and 53BP1 (Bethyl A300-272A), and secondary antibodies: Goat anti-mouse IgG Alexa Fluor 488 (Invitrogen A-11001) and Goat anti-rabbit IgG Alexa Fluor 488 (Invitrogen A-11008), respectively. Slides were imaged using a Deltavision microscope with SoftWorx 6.5.2 software, and images were analyzed using ImageJ 1.53a software.

### DNA fiber combing assays

Cells were incubated with 100 μM IdU and 100 μM CldU as indicated. Chemical compounds were added according to labeling schemes presented. Cells were then collected and processed using the FiberPrep kit (Genomic Vision EXT-001) according to the manufacturer’s instructions. Samples were added to combing reservoirs containing MES solution (2-(N-morpholino) ethanesulfonic acid), and DNA molecules were stretched onto coverslips (Genomic Vision COV-002-RUO) using the FiberComb Molecular Combing instrument (Genomic Vision MCS-001). For S1 nuclease assays, MES solution was supplemented with 1 mM zinc acetate and either 40 U/ml S1 nuclease (ThermoFisher 18 001 016) or S1 nuclease dilution buffer as control and incubated for 30 min at room temperature. Slides were then stained with antibodies detecting CldU (Abcam 6236) and IdU (BD 347580) and incubated with secondary AF488 (Abcam 150 117) or Cy5 (Abcam 6565) conjugated antibodies. Finally, the cells were mounted onto coverslips and imaged using a confocal microscope (Leica SP5) and analyzed using LASX 3.5.7.23225 software.

### SIRF (*in situ* analysis of protein interactions at DNA replication forks) assays

Cells were seeded into 8-chamber slides, and 24 h later they were pulse-labeled with 50 μM EdU and treated with 0.4 mM HU, according to the labeling schemes presented. Cells were permeabilized with 0.5% Triton for 10 min at 4°C, washed with PBS, fixed at room temperature with 4% paraformaldehyde in PBS for 10 min, washed again in PBS and then blocked in 3% BSA in PBS for 30 min. Cells were then subjected to Click-iT reaction with biotin-azide using the Click-iT Cell Reaction Buffer Kit (ThermoFisher C10269) for 30 min and incubated overnight at 4°C with primary antibodies diluted in PBS with 3% BSA. The primary antibodies used were: Biotin (mouse: Jackson ImmunoResearch 200–002-211; rabbit: Bethyl Laboratories A150-109A), MRE11 (GeneTex GTX70212), H3 (Cell Signaling Technology 4499s), CHAF1A (Cell Signaling Technology 5480s) and PrimPol (Proteintech 29824–1-AP). Next, samples were subjected to a proximity ligation reaction using the Duolink kit (MilliporeSigma DUO92008) according to the manufacturer’s instructions. Slides were imaged using a Deltavision microscope with SoftWorx 6.5.2 software, and images were analyzed using ImageJ 1.53a software. To account for variation in EdU uptake between samples, for each sample, the number of nuclear protein–biotin foci were normalized to the average number of nuclear biotin–biotin foci for that respective sample resulting in a comparison of fold change across samples as demonstrated below. The result of this calculation for each nucleus quantified was then averaged and reported.


\begin{eqnarray*}\frac{{\# \, of\, protein - biotin\, foci }}{{Average\, \# \, of\, biotin - biotin\, foci}} &=& relative\, \# \, of\, protein{-}biotin\, foci\end{eqnarray*}

### Statistics and reproducibility

For CellTiterGlo, clonogenic survival assays, SIRF and immunofluorescence, the *t*-test (two-tailed, unpaired) was used. For DNA fiber assays and comet assays, the Mann–Whitney statistical test (two-tailed) was performed. For the clonogenic survival assay testing the effect of ASF1A depletion in BRCA2^KO^ HeLa cells’ response to cisplatin, a two-way Analysis of Variance (ANOVA) significance test was utilized. For immunofluorescence, DNA fiber combing, SIRF and comet assays, results from one experiment are shown; the results were reproduced in at least one additional independent biological conceptual replicate. Statistical analyses were performed using GraphPad Prism 10 and Microsoft Excel v2205 software. Statistical significance is indicated for each graph (ns = not significant, for *P* > 0.05; * for *P* ≤ 0.05; ** for *P* ≤ 0.01; *** for *P* ≤ 0.001, **** for *P* ≤ 0.0001). The source data underlying each of the main and supplementary figure panels, including the values plotted in graphs, the exact *P*-values, and the uncropped blots is presented in the Supplementary Table S1.

## Results

### Identification of genes necessary for survival of CHAF1A^KO^ 293T cells through genome wide-loss-of-function CRISPR screening

**Figure 1.**
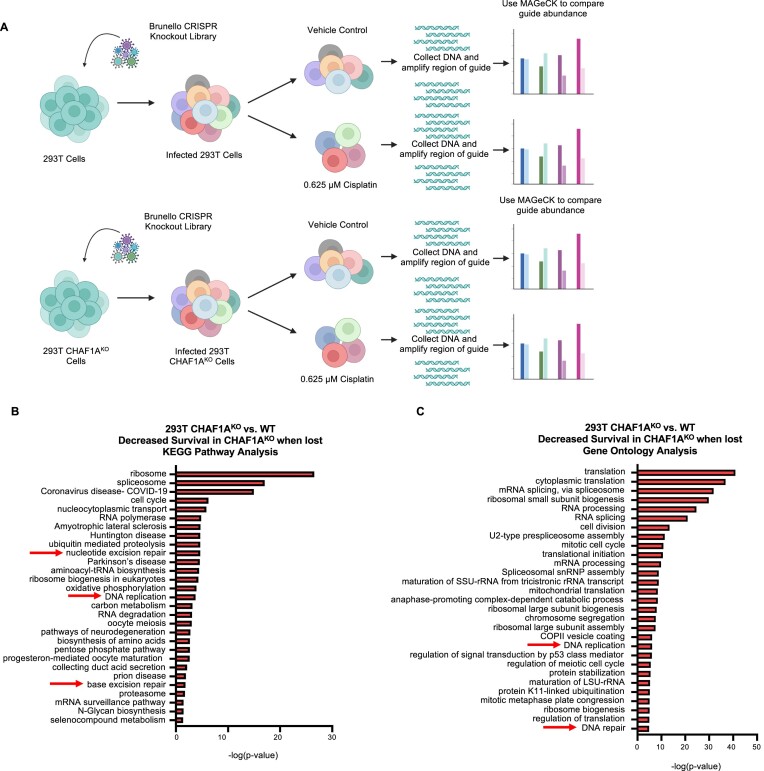
Identification of genes necessary for survival of CHAF1A^KO^ 293T cells through genome wide-loss-of-function CRISPR screening. (**A**) Schematic representation of the CHAF1A^KO^ CRISPR Screen to identify genes required for survival of CHAF1A^KO^ cells. 293T-WT and 293T-CHAF1A^KO^ cells were incubated with and without 0.625 μM cisplatin for 3 days before being collected and analyzed via MAGeCK. Created in BioRender. Moldovan, G. (2024) BioRender.com/j16o386. (**B** and **C**) Overrepresentation analyses of biological pathways of the top hits with *P* < 0.01 that result in decreased cell survival in 293T-CHAF1A^KO^ cells compared to 293T-WT cells. KEGG (B) and Gene Ontology (C) terms with negative log*P* >1.40 and 4.85, respectively, are presented.

We previously showed that loss of CHAF1A results in a defect in protection of reversed replication forks in HR proficient cells under replication stress (25). Because FP may be a determinant of responsiveness to cisplatin, we sought to identify additional factors that could modulate CAF-1’s roles and potentially identify new targets or biomarkers to increase effectiveness of genotoxic chemotherapies. We performed a genome-wide CRISPR genetic screen in CHAF1A-knockout (CHAF1A^KO^) 293T cells (Supplementary Figure S1A) previously established in our lab (25) and control 293T WT cells both untreated and in the presence of cisplatin (Figure 1A). Both cell lines were infected with the Brunello genome-wide CRISPR-knockout lentiviral library which targets 19 114 genes utilizing an average of four gRNAs per gene. To ensure 250-fold coverage of the library, 20 million library infected cells per arm were either untreated or treated with 0.625 μM cisplatin for 3 days before collection. Genomic DNA was isolated and used in a PCR reaction to amplify the gRNA region which was then identified by Illumina sequencing. Multiple MAGeCK analyses were used to create rankings of genes that were less abundant in CHAF1A^KO^ cells compared to WT in untreated and cisplatin treated conditions, as well as to compare gene abundance between untreated and cisplatin treated 293T WT cells (Supplementary Tables S2, S3 and S4).

Overrepresentation analysis of biological pathways of the top hits which, when inactivated, cause decreased survival in CHAF1A^KO^ cells compared to WT in untreated conditions via KEGG, and Gene Ontology networks (735 genes with *P* < 0.01) showed that multiple DNA repair related processes are important in maintaining CHAF1A^KO^ cell survival (Figure 1B and C). These overrepresented pathways include base excision repair, and DNA replication and repair (Figure 1B and C). The other way around, we also analyzed the pathway representation of top hits which cause increased survival in CHAF1A^KO^ cells compared to WT (442 genes with *P* < 0.02). Apoptotic pathways were hyper-represented in these analyses, suggesting that CHAF1A-depleted cells undergo increased apoptosis (Supplementary Figure S1B and C). Interestingly, when we performed the same analysis for cisplatin-treated cells (386 genes with *P* < 0.02), we observed a different pattern, with translation, transcription, replication and cell cycle processes as overrepresented pathways (Supplementary Figure S1D and E). Finally, we also performed analyses of cisplatin sensitivity in 293T WT cells (376 genes with *P*< 0.02), revealing interstrand crosslink repair as a pathway that is more commonly lost in cells sensitive to cisplatin treatment (Supplementary Figure S1F and G). This occurrence should be expected as inter-strand crosslink repair is a known method of cisplatin resistance (32).

**Figure 2.**
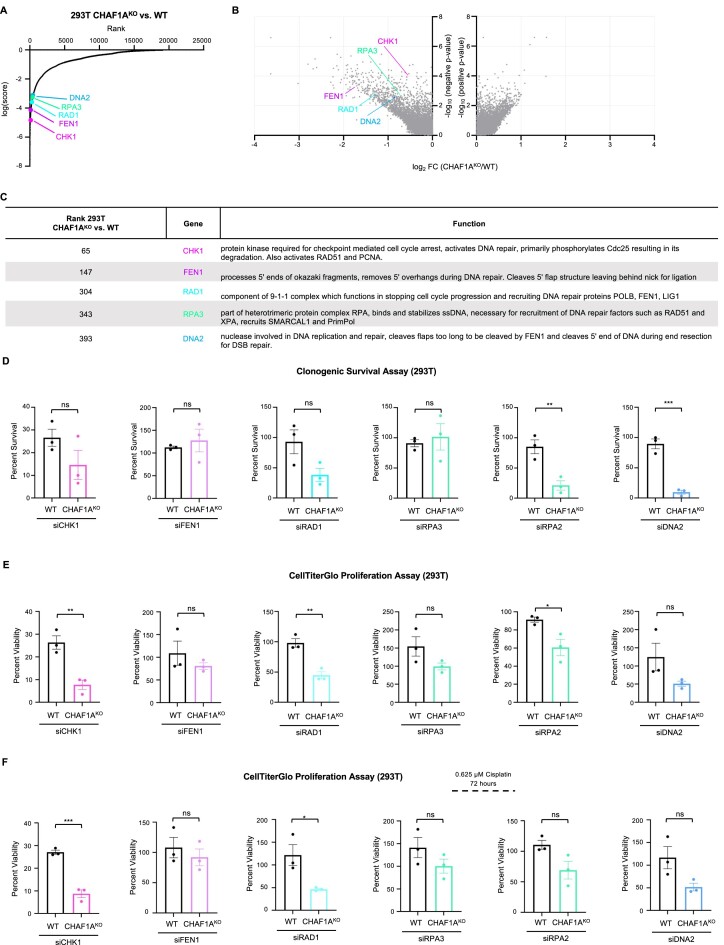
Validation of Top Hits from CRISPR screen to identify necessary genes for CHAF1A^KO^ 293T cell survival. (**A**) Scatterplot showing results of genome-wide CRISPR screen to identify necessary genes for CHAF1A^KO^ 293T cell survival. Genes targeted by the library are ranked according to MAGeCK score which represents the reduced survival of CHAF1A^KO^ 293T cells when lost compared to when lost in 293T control cells. Genes highlighted are those chosen for validation of the screen results. (**B**) Volcano plot showing results of genome-wide CRISPR screen to identify genes necessary for CHAF1A^KO^ 293T cell survival. Genes targeted by the library are plotted by the −log10 of their respective negative and positive *P*-values and associated log2 fold change values with genes clustering to the upper left of the graph demonstrating a reduction in relative survival when lost in the CHAF1A^KO^ 293T cells while those clustering in the upper right of the graph demonstrate an increase in relative survival when lost in the CHAF1A^KO^ 293T cells. Genes highlighted are those chosen for validation of the screen results. (**C**) Table showing ranks and biological functions of selected DNA repair related top hits from screen comparison showing top genes that when lost result in a decrease in survival of CHAF1A^KO^ 293T cells compared to 293T WT cells in the absence of treatment (293T CHAF1A^KO^ NT versus 293T WT NT). (**D**) Clonogenic survival assay showing that depletion of selected top hits in CHAF1A^KO^ 293T cells decreases cell survival compared to WT 293T cells. The sensitivity is presented normalized to respective cell lines transfected with the siRNA negative control. The average of three independent experiments and the SEM indicated as error bars is shown. Asterisks indicate statistical significance (*t*-test unpaired). (**E**) CellTiterGlo proliferation assay showing that depletion of selected top hits in CHAF1A^KO^ 293T cells decreases cell viability compared to WT 293T cells. Viability is presented normalized to respective cell lines transfected with the siRNA negative control. The average of three independent experiments and the SEM indicated as error bars is shown. Asterisks indicate statistical significance (*t*-test unpaired). (**F**) CellTiterGlo proliferation assay showing that depletion of CHK1 and RAD1 in CHAF1A^KO^ cells treated with 0.625 μM cisplatin for 3 days decreases cell viability compared to 293T WT cells. Viability is presented normalized to respective cell lines transfected with the siRNA negative control. The average of three independent experiments and the SEM indicated as error bars are shown. Asterisks indicate statistical significance (*t*-test unpaired).

As multiple DNA repair pathways are present in our top hits (735 genes with *P* < 0.01) that lead to cell death in CHAF1A^KO^ cells as compared to WT, we searched our lists for DNA repair genes on which to focus our exploration of finding targets that influence CAF-1 function (Figure 2A and B). We identified the cell cycle/checkpoint proteins CHK1 and RAD1, as well as the DNA replication and repair proteins FEN1, RPA3 and DNA2 as top hits of interest which when lost lead to increased cell death in CHAF1A^KO^ cells (Figure 2C).

### Validation and functional investigation of top hits from CRISPR screen identifying genes required for 293T-CHAF1A^KO^ cell survival

After selecting these top hits of interest from the screen results, we sought to validate the effects of loss of these genes in the 293T-CHAF1A^KO^ cells, using both clonogenic survival assays and CellTiterGlo viability assay. Since RPA3 is a component of the Replication Protein A (RPA) complex, we also included in our analyses the RPA2 subunit. With the notable exception of FEN1 and RPA3, we generally observed a greater reduction in cellular viability upon siRNA-mediated knockdown of these genes in CHAF1A^KO^ cells compared to WT 293T cells, in both clonogenic survival assays (Figure 2D) and CellTiterGlo proliferation assay (Figure 2E). Moreover, we obtained similar results when treating cells with 0.625 μM cisplatin for 3 days (Figure 2F). Western blots confirmed the siRNA-mediated knockdown of the genes investigated (Supplementary Figure S2A). Overall, these results validate the CRISPR screens performed.

**Figure 3.**
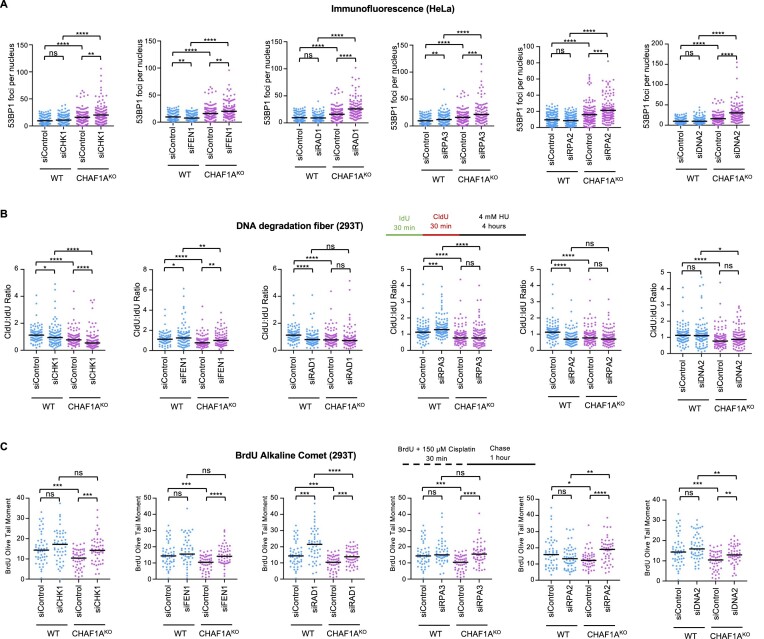
Functional investigation of Top Hits from CRISPR screen to identify necessary genes for CHAF1A^KO^ 293T cell survival. (**A**) 53BP1 immunofluorescence showing that depletion of selected top hits in CHAF1A^KO^ 293T cells result in an increase in 53BP1 foci induction compared to 293T WT cells in the absence of exogenous DNA damage. At least 129 cells were quantified for each condition. The mean value is represented on the graphs, and asterisks indicate statistical significance (*t*-test two-tailed, unpaired). (**B**) DNA fiber assay to evaluate fork degradation showing differing impacts between the selected top hits when depleted in CHAF1A^KO^ 293T cells. Depletion of CHK1 results in a significant increase in fork degradation, while depletion of FEN1 leads to an incomplete rescue of FP in CHAF1A^KO^ cells. Depletion of RAD1 and RPA2 demonstrated no change in degradation in CHAF1A^KO^ cells despite a loss of FP in WT cells, while depletion of RPA3 and DNA2 did not alter FP status in either cell line. At least 100 fibers were quantified per condition. The ratio of CldU to IdU tract lengths is presented with median values marked. Asterisks indicate statistical significance (Mann–Whitney test, two-tailed). Schematic representations of the DNA fiber combing assay conditions are presented. (**C**) BrdU alkaline comet assay showing that depletion of selected top hits besides RAD1 result in an increase in ssDNA gap accumulation in CHAF1A^KO^ 293T cells compared to 293T WT cells upon treatment with 150 μM cisplatin. At least 50 nuclei were quantified per condition with median values marked on the graph. Asterisks indicate statistical significance (Mann–Whitney, two-tailed). A schematic representation of the assay conditions is displayed.

Since CAF-1 is involved in DNA repair, we next investigated if this decrease in survival observed upon knockdown of the top hits in CHAF1^KO^ cells is associated with increased DNA damage. To this end we measured spontaneous 53BP1 foci formation as a marker of DSB break formation. Depletion of any of the top hits resulted in an increase in 53BP1 foci formation in CHAF1A^KO^ cells (Figure 3A). Moreover, we conducted neutral comet assays to measure DSB formation after depleting the selected top hits in WT cells and CHAF1A^KO^ cells treated with 150 μM cisplatin for 2 h. Generally, depletion of the top hits of interest in the CHAF1A^KO^ cells resulted in an increase in DSB formation to a larger extent than in WT cells (Supplementary Figure S3A). Increased DSB formation was observed for FEN1 as well, even though it appears to not affect cellular viability. This surprising results possibly reflects the many roles of FEN1 in genome stability, which may result in conflicting phenotypes.

As we previously showed that loss of CHAF1A results in a defect in replication FP (9,25), we tested if degradation of reversed replication forks was further exacerbated in CHAF1A^KO^ cells when top hits were depleted. We measured fork degradation using the DNA fiber combing assay. We incubated cells with thymidine analogs IdU and CldU followed by treatment with 4 mM HU to induce fork arrest and reversal. As we previously showed (25), CHAF1A^KO^ cells showed a reduction in CldU:IdU ratios compared to WT cells, indicating a FP defect. Interestingly, only depletion of CHK1 resulted in a further increase in replication fork degradation in CHAF1A^KO^ cells, as observed by a decrease in the CldU:IdU ratios (Figure 3B). We previously showed that CAF-1 becomes trapped at lagging strand gaps in inactive replication complexes behind ongoing forks in BRCA2-depleted cells, which may contribute to fork degradation (25). While not statistically significant, loss of CHK1 caused an increase in CHAF1A retention under these conditions (Supplementary Figure S3B). Other than CHK1, the rest of the top hits shared one of two patterns in the effect on replication fork degradation: either knock-down of the top hit resulted in an increase in degradation in the WT cells and had no effect on CHAF1A^KO^ cells, or knockdown of the top hit has no effect on replication fork degradation in either cell line (Figure 3B). Overall, these findings argue that, in general, increased fork degradation does not explain the reduction in cell proliferation observed upon depletion of the top hits in CHAF1A^KO^ cells.

We next sought to investigate if, rather than a FP defect, the failure to suppress the accumulations of other replication-associated lesions, such as ssDNA gaps, explain the decrease in survival upon depletion of the top hits in CHAF1A^KO^ cells, particularly since recent studies have shown that suppression of ssDNA gaps correlates with chemosensitivity (4,16). To address this, we employed the BrdU alkaline comet assay which can detect, among other lesions, replication-associated ssDNA gaps (33–37). Upon treating cells with 150μM cisplatin for 30 min, we observed that depletion of CHK1, FEN1, RPA3, RPA2 and DNA2 resulted in a relative increase in the olive tail moment in CHAF1A^KO^ cells, while depletion of RAD1 resulted in a larger increase in ssDNA gaps in WT cells (Figure 3C). These findings potentially suggest that ssDNA gap accumulation may account for the increased sensitivity observed upon depletion of the top hits in CHAF1A^KO^ cells.

### Loss of CHAF1A leads to suppression of nascent strand ssDNA gaps

**Figure 4.**
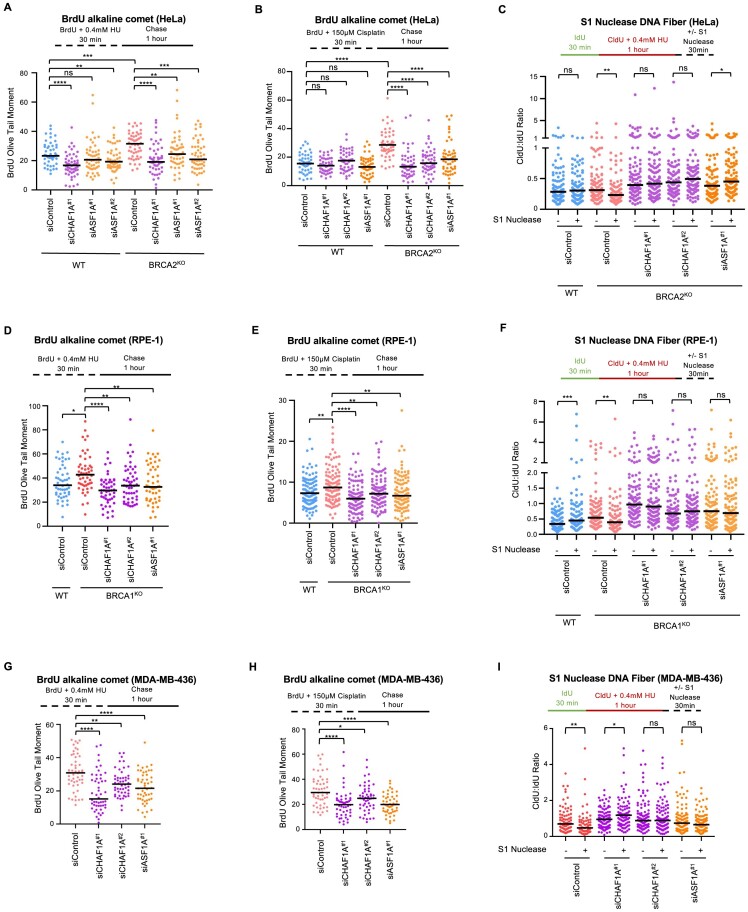
Loss of CHAF1A leads to suppression of ssDNA gaps. (**A** and **B**) BrdU alkaline comets showing that depletion of CHAF1A or ASF1A in WT or BRCA2^KO^ HeLa cells results in a decrease in ssDNA gap accumulation upon treatment with (A) 0.4 mM HU or (B) 150 μM cisplatin. At least 49 nuclei were quantified per condition with median values marked on the graph. Asterisks indicate statistical significance (Mann–Whitney, two-tailed). Schematic representations of the assay conditions are displayed. Western blot confirming CHAF1A and ASF1A depletion are shown in Supplementary Figure S4A. (**C**) S1 Nuclease DNA fiber combing assay showing that CHAF1A or ASF1A depletion in BRCA2^KO^ HeLa cells results in a decrease in ssDNA gap accumulation upon treatment with 0.4 mM HU. At least 87 fibers were quantified per condition. The ratio of CldU to IdU tract lengths is presented with median values marked. Asterisks indicate statistical significance (Mann–Whitney test, two-tailed). A schematic representation of the DNA fiber combing assay conditions is displayed. (**D** and **E**) BrdU alkaline comets showing that depletion of CHAF1A or ASF1A decreases ssDNA gaps in RPE1 BRCA1^KO^ cells in a similar manner to their depletion in BRCA2-deficient cells when treated with (D) 0.4 mM HU or (E) 150 μM cisplatin. At least 50 nuclei were quantified per condition with median values marked on the graph. Asterisks indicate statistical significance (Mann–Whitney, two-tailed). Schematic representations of the assay conditions are displayed. (**F**) S1 Nuclease DNA fiber combing assay showing that CHAF1A or ASF1A depletion in BRCA1^KO^ RPE1 cells results in a decrease in ssDNA gap accumulation upon treatment with 0.4 mM HU. At least 100 fibers were quantified per condition. The ratio of CldU to IdU tract lengths is presented with median values marked. Asterisks indicate statistical significance (Mann–Whitney test, two-tailed). A schematic representation of the DNA fiber combing assay conditions is displayed. (**G** and **H**) BrdU alkaline comets showing that depletion of CHAF1A or ASF1A in MDA-MB-436 cells results in a decrease in ssDNA gap accumulation upon treatment with (G) 0.4 mM HU or (H) 150 μM cisplatin. At least 50 nuclei were quantified per condition with median values marked on the graph. Asterisks indicate statistical significance (Mann–Whitney, two-tailed). Schematic representations of the assay conditions are displayed. (**I**) S1 Nuclease DNA fiber combing assay showing that CHAF1A or ASF1A depletion in MDA-MB-436 cells results in a decrease in ssDNA gap accumulation upon treatment with 0.4 mM HU. At least 66 fibers were quantified per condition. The ratio of CldU to IdU tract lengths is presented with median values marked. Asterisks indicate statistical significance (Mann–Whitney test, two-tailed). A schematic representation of the assay conditions is displayed.

Unexpectedly, we observed that even without any gene knockdown, CHAF1A^KO^ cells had less replication-associated ssDNA gaps than WT cells (Figure 3C). We sought to further characterize this phenotype in additional genetic backgrounds and with different genotoxic agents. We performed BrdU alkaline comet assays with depletion of CHAF1A as well as its cooperating histone chaperone ASF1A in HeLa WT and BRCA2^KO^ cells previously generated in our lab (26). Knockdown of CHAF1A or ASF1A in either WT or BRCA2 deficient HeLa cells resulted in a marked decrease in ssDNA gap accumulation after treatment with either 0.4 mM HU (Figure 4A and Supplementary Figure S4A) or 150 μM cisplatin (Figure 4B). We validated these findings using a more specific assay to measure nascent strand ssDNA gaps, namely the S1 nuclease DNA fiber combing assay. As we previously showed (33), HeLa-BRCA2^KO^ cells show ssDNA gap accumulation upon treatment with 0.4 mM HU, as evidenced by a decrease in CldU:IdU ratios in S1-treated samples compared to non-S1-treated samples (Figure 4C and Supplementary Figure S4B). Depletion of CHAF1A or of ASF1A suppressed this ssDNA gap accumulation in HeLa-BRCA2^KO^ cells. We next investigated if this phenotype is restricted to BRCA2-deficient cells. To this end, we employed BRCA1-knockout RPE1 cells. Knockdown of CHAF1A or of ASF1A suppressed ssDNA gap accumulation in these cells, in both the BrdU alkaline and S1 nuclease DNA fiber combing assays (Figure 4D–F). Similar findings were observed in BRCA1-mutant patient-derived MDA-MB-436 cells (Figure 4G–I). Additionally, we obtained similar results upon induction of ssDNA gaps by exposure to the PARPi olaparib (Supplementary Figure S4C). Finally, we observed that depletion of BRCA1 in 293T-CHAF1A^KO^ cells failed to elicit the expected increase in ssDNA gap accumulation achieved in WT 293T cells under cisplatin treatment (Supplementary Figure S4D). Overall, these findings indicate that CAF-1 promotes nascent strand gap accumulation.

### Suppression of ssDNA gaps upon CHAF1A loss is independent of H3 deposition at actively replicating DNA

**Figure 5.**
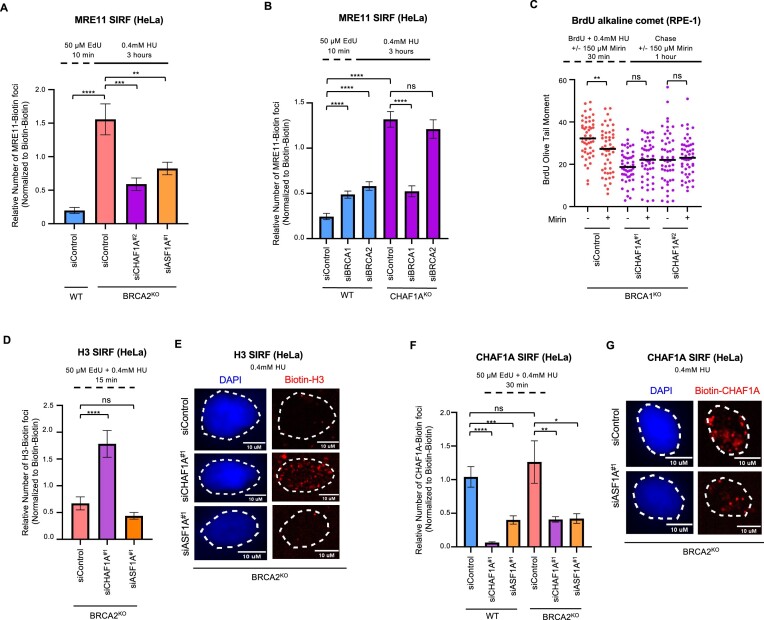
Suppression of ssDNA gaps upon CHAF1A loss is independent of H3 deposition at actively replicating DNA. (**A**) SIRF assay for MRE11 localization showing that BRCA2^KO^ induced localization of MRE11 to nascent DNA under 0.4 mM HU treatment is abrogated upon depletion of CHAF1A or ASF1A in BRCA2^KO^ HeLa cells. At least 75 cells were quantified for each condition. Bars indicate the mean values, error bars represent SEM, and asterisks indicate statistical significance (*t*-test, two-tailed, unpaired). A schematic representation of the assay conditions is shown. (**B**) SIRF assay showing that increased MRE11 recruitment to nascent DNA in response to knockdown of BRCA1 and BRCA2 in WT 293T cells is no longer present when CHAF1A is lost in CHAF1A^KO^ 293T cells under 0.4 mM HU treatment. At least 186 cells were quantified for each condition. Bars indicate the mean values, error bars represent SEM, and asterisks indicate statistical significance (*t*-test, two-tailed, unpaired). A schematic representation of the assay conditions is shown. (**C**) BrdU alkaline comet assay showing that inhibition of MRE11 via 150 μM mirin treatment is epistatic with the decrease in ssDNA gap accumulation observed with knockdown of CHAF1A or ASF1A in BRCA1^KO^ RPE1 cells. At least 50 nuclei were quantified per condition with median values marked on the graph. Asterisks indicate statistical significance (Mann–Whitney, two-tailed). Cells were incubated with or without mirin during the BrdU incubation period as well as with their respective mirin conditions during the following 1 h chase as shown in the schematic representation of the assay conditions displayed. (**D** and **E**) SIRF experiment showing differential impact of CHAF1A and ASF1A depletions on H3 localization to nascent DNA. Knockdown of CHAF1A in BRCA2^KO^ HeLa cells leads to a rescue of H3 localization to nascent DNA under 0.4 mM HU treatment, whereas knockdown of ASF1A under these same conditions result in no change from control (D). Representative micrographs with scale bars representing 10 μm are shown (E). At least 100 cells were quantified for each condition. Bars indicate the mean values, error bars represent SEM, and asterisks indicate statistical significance (*t*-test, two-tailed, unpaired). A schematic representation of the assay conditions is shown. (**F** and **G**) SIRF assay showing that CHAF1A localization to nascent DNA is decreased after treatment with 0.4 mM HU upon ASF1A knockdown in both WT and BRCA2^KO^ HeLa cells similarly to those with CHAF1A knocked down (F). Representative micrographs with scale bars representing 10 μm are shown (G). At least 100 cells were quantified for each condition. Bars indicate the mean values, error bars represent SEM, and asterisks indicate statistical significance (*t*-test, two-tailed, unpaired). A schematic representation of the assay conditions is shown.

Nucleases such as MRE11, EXO1 and DNA2 have recently been shown to carry out expansion of ssDNA gaps in BRCA-deficient cells (2,8,33,38). We thus investigated the role of MRE11 in the accumulation of CAF-1-dependent ssDNA gaps. We first used the SIRF assay (*in situ* analysis of protein interactions at DNA replication forks) to measure the recruitment of MRE11 to nascent DNA under ssDNA gap-inducing conditions (33). As expected, BRCA2^KO^ cells showed increased MRE11 on nascent DNA after treatment with 0.4 mM HU (Figure 5A). Loss of CHAF1A results in a decrease in MRE11 SIRF signal, in line with the reduction in gap formation observed under these conditions. Similarly, MRE11 localization increased upon BRCA1 or BRCA2 depletion in WT cells, but not when BRCA1 or BRCA2 are depleted in CHAF1A^KO^ cells (Figure 5B and Supplementary Figure S5A). In the absence of BRCA deficiency, CHAF1A^KO^ cells showed higher levels of MRE11 background binding to DNA, potentially reflecting the previously described impact of chromatin compaction on suppressing the DNA accessibility of MRE11 (39–41). We next examined the impact of the MRE11 inhibitor mirin on the ssDNA gap suppression observed upon CHAF1A or ASF1A loss in BRCA deficient cells. Treatment with mirin was epistatic with CHAF1A or ASF1A loss as no further suppression of replication-associated DNA lesions was observed in cells that already had lost the histone chaperone (Figure 5C). Inhibition of DNA2 by the specific inhibitor C5 had a similar effect (Supplementary Figure S5B). Taken together, these findings show that MRE11 localization to ssDNA gaps correlates with the gap accumulation observed in CAF-1 proficient cells.

Nucleosome presence on DNA has been shown to modulate nucleolytic capabilities of these nucleases (39,40,42,43). It is possible that, when high amounts of histones are deposited at actively replicating DNA, nucleases would be less effective in their expansion of ssDNA gaps and thus less ssDNA gaps would be present. CAF-1′s role in modulating ssDNA gap abundance may thus reflect its histone deposition function, in that increased nucleosome density at replication forks could be hindering nuclease expansion of ssDNA gaps. To address this, we sought to investigate the deposition of histone H3 on nascent DNA under ssDNA gap-inducing conditions. Histone H3 SIRF experiments showed that loss of CHAF1A or of ASF1A reduced H3 levels on nascent DNA (Supplementary Figure S5C), as expected from the roles of these histone chaperones in histone deposition. In BRCA2-knockout cells, we previously showed that histone deposition is defective because of CAF-1 retention on inactive complexes away from ongoing replication forks (25). However, loss of CAF-1 releases a compensatory histone deposition pathway, involving ASF1 and the Histone Cell Cycle Regulator (HIRA) complex, which restores histone deposition. In line with this, depletion of CHAF1A increased H3 levels in BRCA2-knockout cells under gap-forming conditions (Figure 5D,E). In contrast, loss of ASF1A did not show an increase, in line with our previous findings that ASF1 is required for the HIRA compensatory histone deposition pathway. Since loss of CAF-1 and loss of ASF1 show similar suppression of gap accumulation but differences in the H3 levels on nascent DNA, these findings suggest that gap accumulation occurs in a manner independent of histone deposition.

Instead, we considered that alternative possibility that CAF-1 promotes gap formation in a manner which does not involve its nucleosome deposition function but requires its localization to nascent DNA. Indeed, SIRF experiments showed that CHAF1A localizes to nascent DNA under gap-inducing conditions (Figure 5F and G). As control, CHAF1A depletion reduced the SIRF signal, showing the specificity of the signal observed. Since ASF1 loss also caused ssDNA gap accumulation, we investigate its impact on CAF-1 localization. ASF1A knockdown reduced the CHAF1A SIRF signal similarly to CHAF1A knockdown (Figure 5F and G). This is in line with a recent study showing that efficient CAF-1 interaction with PCNA depends on prior H3-H4 dimer binding to the complex (19). Thus, unavailability of H3-H4 dimers may impair CAF-1 recruitment to nascent DNA, explaining why loss of ASF1A inhibits this recruitment. Since loss of CHAF1A and loss of ASF1A differentially impact nucleosome deposition but similarly affect CAF-1 presence on nascent DNA, these findings indicate that the CAF-1–ASF1 pathway does not influence ssDNA gap accumulation in an H3 deposition-dependent manner, but rather CAF-1 localization to the replication fork under conditions of replication stress may be important.

### CAF-1 promotes PrimPol recruitment to nascent DNA

**Figure 6.**
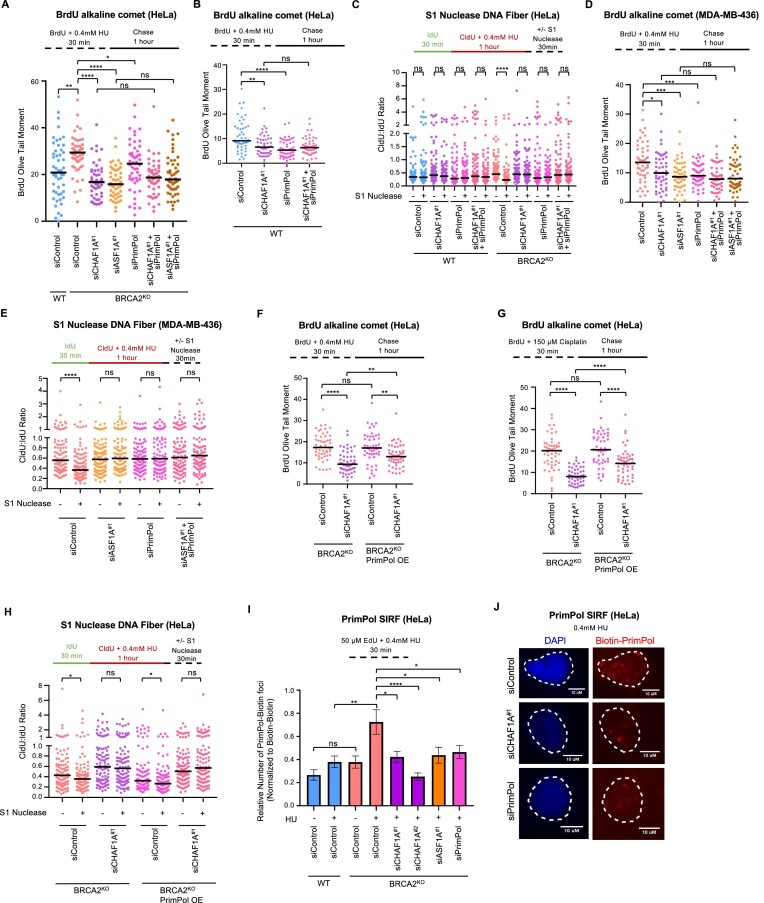
CHAF1A depletion is epistatic with PrimPol depletion and results in a decrease in PrimPol localization to replication forks. (**A** and **B**) BrdU alkaline comet assay showing that loss of PrimPol is epistatic with loss of either CHAF1A or ASF1A in (A) BRCA2^KO^ or (B) WT HeLa cells. Accumulation of ssDNA gaps in samples depleted of either CHAF1A or ASF1A show no further decrease in ssDNA gaps when PrimPol is also depleted. At least 49 nuclei were quantified per condition with median values marked on the graph. Asterisks indicate statistical significance (Mann–Whitney, two-tailed). Schematic representations of the assay conditions are shown. (**C**) S1 Nuclease DNA fiber combing assay showing that CHAF1A co-depletion with PrimPol in WT or BRCA2KO HeLa cells does not result in a further decrease in ssDNA gap accumulation upon treatment with 0.4 mM HU. At least 100 fibers were quantified per condition. The ratio of CldU to IdU tract lengths is presented with median values marked. Asterisks indicate statistical significance (Mann–Whitney test, two-tailed). A schematic representation of the assay conditions is displayed. (**D**) BrdU alkaline comet assay showing that loss of PrimPol is epistatic with loss of either CHAF1A or ASF1A in MDA-MB-436 cells. Accumulation of ssDNA gaps in samples depleted of either CHAF1A or ASF1A show no further decrease in ssDNA gaps when PrimPol is also depleted. At least 50 nuclei were quantified per condition with median values marked on the graph. Asterisks indicate statistical significance (Mann–Whitney, two-tailed). A schematic representation of the assay conditions is displayed. (**E**) S1 Nuclease DNA fiber combing assay showing that CHAF1A or ASF1A co-depletion with PrimPol in MDA-MB-436 cells does not result in a further decrease in ssDNA gap accumulation upon treatment with 0.4 mM HU. At least 100 fibers were quantified per condition. The ratio of CldU to IdU tract lengths is presented with median values marked. Asterisks indicate statistical significance (Mann–Whitney test, two-tailed). A schematic representation of the assay conditions is displayed. (**F** and **G**) BrdU alkaline comets showing the overexpression of PrimPol in BRCA2^KO^ HeLa cells under 0.4 mM HU (F) and 150 μM cisplatin (G) treatment is able to partially rescue ssDNA gap abundance when CHAF1A is knocked down. At least 50 nuclei were quantified per condition with median values marked on the graph. Asterisks indicate statistical significance (Mann–Whitney, two-tailed). Schematic representations of the assay conditions are displayed. (**H**) S1 Nuclease DNA fiber combing assay showing that overexpression of PrimPol in BRCA2^KO^ HeLa cells is not able to rescue ssDNA gap accumulation to an observable level upon treatment with 0.4 mM HU. At least 100 fibers were quantified per condition. The ratio of CldU to IdU tract lengths is presented with median values marked. Asterisks indicate statistical significance (Mann–Whitney test, two-tailed). A schematic representation of the assay conditions is displayed. (**I** and **J**) SIRF experiment showing that the increase in PrimPol localization to nascent DNA upon 0.4 mM HU treatment in BRCA2^KO^ HeLa cells is lost when either CHAF1A or ASF1A are depleted (I). Representative micrographs with scale bars representing 10 μm are shown (J). At least 100 cells were quantified for each condition. Bars indicate the mean values, error bars represent SEM, and asterisks indicate statistical significance (*t*-test, two-tailed, unpaired). A schematic representation of the assay conditions is shown.

Recently, CHAF1A was shown to contain an RPA2 binding motif and it was proposed that CHAF1A interaction with RPA2 was responsible for mediating RAD18 recruitment to RPA under replication stress (44). Since the findings described above suggested that CAF-1 may have a role in the generation of ssDNA gaps rather than the expansion of ssDNA gaps, we speculated that perhaps CHAF1A acts in a similar manner to mediate PrimPol recruitment to RPA. To test this, we first investigated if PRIMPOL and the CAF-1–ASF1 pathway are epistatic. Co-depletion of CHAF1A or ASF1A and PrimPol in HeLa-WT, HeLa-BRCA2^KO^ and MDA-MB-436 cells showed no further decrease in ssDNA gap accumulation in both BrdU alkaline comet and S1 nuclease DNA fiber combing assays, implying that the ssDNA gaps dependent on the histone chaperones that are lost are at least partially generated by PrimPol (Figure 6A–E and Supplementary Figure S6A and B). Next, using a BRCA2^KO^ HeLa cell line overexpressing PrimPol previously generated in our laboratory (27), we analyzed whether ssDNA gap formation could be restored by increasing the amount of PrimPol available. Under both HU and cisplatin treatment, overexpression of PrimPol was able to partially restore ssDNA gap formation when CHAF1A was depleted (though not to normal levels) (Figure 6F–H and Supplementary Figure S6C). This indicates that increased PrimPol expression can bypass the need for the CAF-1–ASF1 pathway in gap formation.

To directly assess whether PrimPol localization could be impaired by CHAF1A loss, we measured PrimPol recruitment to the replication fork using a PrimPol SIRF assay. We were able to detect PrimPol recruitment to nascent DNA upon treatment with 0.4 mM HU (Figure 6I and J). As expected, this recruitment was increased in BRCA2^KO^ cells. PrimPol depletion reduced the SIRF foci observed, confirming the specificity of the signal. Decreases in PrimPol SIRF signal were observed when either CHAF1A or ASF1A were lost, implying that PrimPol recruitment to nascent DNA is dependent on both CHAF1A and ASF1A (Figure 6I and J; Supplementary Figure S6D). Taken together, these results suggest that the CAF-1–ASF1 pathway promotes PrimPol-dependent ssDNA gaps and that this may occur through CHAF1A recruiting PrimPol to nascent DNA.

### Loss of ASF1A results in chemoresistance in BRCA-deficient cells

**Figure 7.**
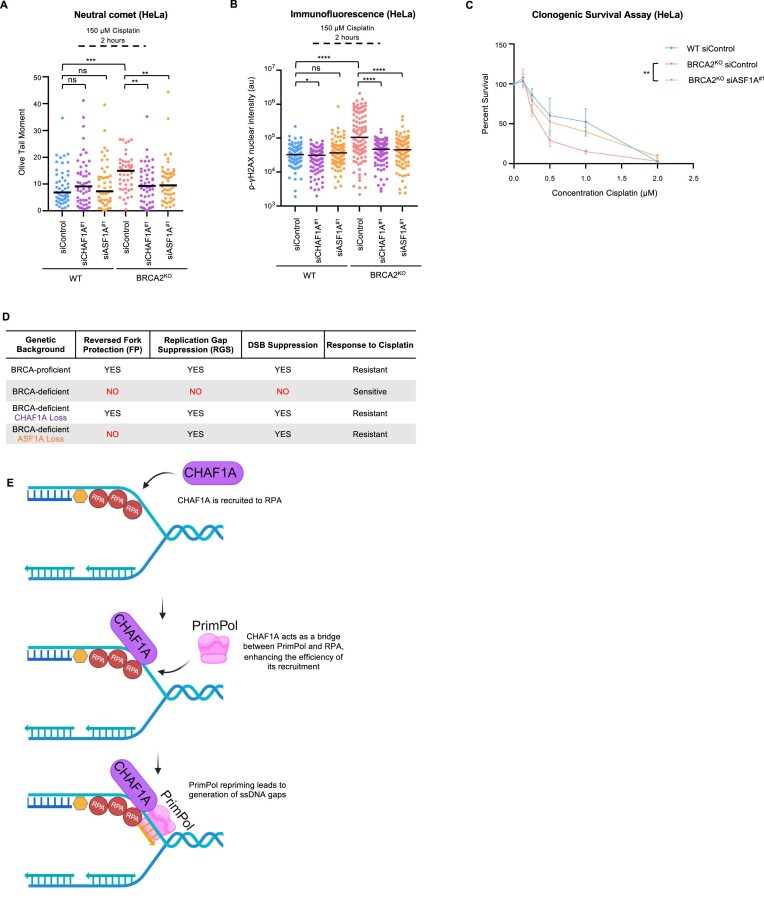
Loss of ASF1A results in chemoresistance in BRCA-deficient cells. (**A**) Neutral comet assay showing that DSBs in BRCA2^KO^ HeLa cells induced by 150 μM cisplatin treatment for 2 h are suppressed with knockdown of ASF1A similarly to knockdown of CHAF1A. At least 47 nuclei were quantified per condition with median values marked on the graph. Asterisks indicate statistical significance (Mann–Whitney, two-tailed). Schematic representations of the assay conditions are displayed. (**B**) γH2AX immunofluorescence showing that γH2AX foci induced by 150 μM cisplatin treatment for 2 h in BRCA2^KO^ HeLa cells are suppressed by knockdown of ASF1A similarly to knockdown of CHAF1A. At least 100 cells were quantified for each condition. The mean value is represented on the graphs, and asterisks indicate statistical significance (*t*-test two-tailed, unpaired). (**C**) Clonogenic survival assay showing that loss of ASF1A promotes cisplatin resistance in BRCA2^KO^ HeLa cells. The average of three experiments, with SEM indicated as error bars, is shown. Asterisks indicate statistical significance (two-way ANOVA). (**D**) Table summarizing the observed consequences of CHAF1A or ASF1A loss in BRCA-deficient cells on FP, RGS, DSB accumulation and drug response status. (**E**) Schematic model of the proposed model of CAF-1 role in efficient PrimPol recruitment to the replication fork and ssDNA gap generation. Created in BioRender. Moldovan, G. (2024) BioRender.com/y25 × 850.

We have previously reported that loss of CHAF1A leads to cisplatin resistance in BRCA deficient cells, ascribing this effect to a restoration of replication FP through a ASF1-HIRA-dependent compensatory mechanism of nucleosome establishment (25). We showed that CHAF1A depletion restored FP in BRCA2^KO^ cells. However, loss of ASF1A did not, since ASF1 is upstream of HIRA in the compensatory histone deposition pathway induced by CAF-1 depletion. Here, we show that both depletion of CHAF1A and of ASF1A restores gap suppression. This situation allows us to differentiate which molecular defect leads to the change in chemosensitivity, by correlating the impact of ASF1A loss on chemosensitivity to its impact on either FP or RGS. Depletion of ASF1A in BRCA2^KO^ HeLa cells showed a decrease in Olive tail moment in a neutral comet assay as well as a decrease in γH2AX nuclear intensity in a γH2AX immunofluorescence assay, implying a reduction in DSBs (Figure 7A and B). Moreover, in a clonogenic survival assay, depletion of ASF1A, like CHAF1A (25), rescued the chemoresistance of BRCA2^KO^ HeLa cells to cisplatin (Figure 7C). Because ASF1A depletion does not affect FP, these results show that cisplatin resistance correlates with RGS rather than FP (Figure 7D). Altogether, our findings suggest that CAF-1 enhances PrimPol localization to nascent DNA and its loss promotes chemoresistance of BRCA-deficient cells to cisplatin, which correlates with suppression of ssDNA gaps (Figure 7E).

## Discussion

Our work suggests that the histone chaperones CAF-1 and ASF1A have unappreciated roles in promoting the accumulation of ssDNA gaps in both HR proficient and deficient cells (Figure 4). Because we previously described a HIRA-dependent compensatory mechanism by which nucleosomes are assembled at replicating DNA when CAF-1 is lost in HR-deficient cells (25), we speculated that loss of ASF1A would render this compensatory establishment mechanism inactive resulting in disparate nucleosome assembly status between CAF-1 and ASF1A-depleted HR-deficient cells. Indeed, we show that depletion of CHAF1A in HR-deficient cells restores nucleosome deposition to the replication fork while ASF1A depletion does not (Figure 5D and E; Supplementary Figure S5C). As the change in nucleosome abundance when CAF-1 or ASF1 are lost does not correlate with the ssDNA gap accumulation status, gap suppression does not seem to be dependent on nucleosome abundance. On the other hand, a recent study indicated that CAF-1-independent changes in chromatin condensation are involved in suppressing ssDNA gap formation (45). Stressed replication forks were shown to be subjected to G9a-mediated chromatin compaction, which suppresses the recruitment of PrimPol and thus gap formation.

A recent study showed that CHAF1A contains a previously unreported RPA2 binding motif at residues 409–418 (44). The study showed that CHAF1A directly interacts with RPA2 and through this interaction is able to serve as a bridge between RPA2 and RAD18, mediating their interaction and enabling RAD18-dependent ubiquitination of PCNA, thus driving TLS (44). We hypothesized that this CHAF1A-mediated recruitment to RPA mechanism could potentially enhance the recruitment of other RPA-binding proteins. Because PrimPol is able to generate ssDNA gaps as a byproduct of its repriming function and is canonically recruited to RPA (46) in order to perform repriming, we suggest that perhaps CHAF1A performs a similar function in enhancing its recruitment thus explaining the observed ssDNA gap promotion phenotype. The exact molecular mechanism remains unclear. Nevertheless, we demonstrate that in BRCA-deficient cells, where PrimPol normally generates an increase in ssDNA gaps in response to genotoxic therapies, loss of CHAF1A or ASF1A decreases PrimPol recruitment to the replication fork (Figure 6I and J), suggesting that these histone chaperones at least enhance its recruitment.

Moreover, to explain the histone deposition-independent impact of ASF1, we showed that CHAF1A recruitment to the replication fork is impaired upon ASF1A loss (Figure 5F and G). This finding agrees with recent data showing that H3-H4 dimers are required for CHAF1A interaction with PCNA (19). This interaction was attributed to a conformational change in PIP, KER, and WHD domains of CHAF1A induced upon H3-H4 binding to its acidic domain. Importantly, the newly reported RPA2 interacting region in CHAF1A coincides with the area of the KER domain (residues 311–445) (47) and could also be conformationally altered by H3-H4 binding. We speculate that CHAF1A and by proxy ASF1A promote PrimPol-dependent ssDNA gap generation through enhancing PrimPol’s recruitment to RPA at stressed replication forks. We further speculate that this effect of ASF1A on PrimPol recruitment occurs through a loss of H3-H4 deliverance to the CAF-1 complex when ASF1A is not present, thus not triggering conformational changes required for its tracking to the replication fork. Overall, these findings build on a recently emerging model of the histone chaperone CAF-1, independent of its histone deposition function, being implicated in promoting various processes of DNA damage tolerance through enhancing recruitment of other DNA repair factors to the replication fork.

We previously reported that, in BRCA-deficient cells, CAF-1 is retained in inactive replication complexes at ssDNA gaps behind ongoing replication forks (25). We showed that loss of PrimPol did not affect CAF-1 levels behind ongoing forks, but inhibition of Polα decreased it. This argues that CAF1 does not become trapped at PrimPol-dependent gaps on the leading strand, but rather at Polα-dependent gaps on the lagging strand. Intriguingly, CHAF1A depletion seemed to result in an increase in fork speed in the presence of HU, in both WT and BRCA-deficient cells (Figure 4C, F and I, and Supplementary Figure S4B). The mechanism underpinning this interesting finding is currently unclear.

We previously reported that CHAF1A loss in HR-deficient cells promotes chemoresistance to cisplatin. We proposed that this occurs through restored FP, since CHAF1A depletion suppressed fork degradation in BRCA-deficient cells (25). Since in the present study we discovered that loss of CHAF1A promotes RGS, it became unclear to what molecular lesion this previously observed chemoresistance is attributable to. To address this, we demonstrate that loss of ASF1A in HR-deficient cells, similar to CHAF1A loss, promotes chemoresistance of HR-deficient cells to cisplatin (Figure 7A–C). Because ASF1A loss does not restore FP but rather promotes RGS, these results suggest that ssDNA gap suppression is at least a contributing driver of chemoresistance when these histone chaperones are lost in HR-deficient cells. We recently showed that unrepaired ssDNA gaps are ultimately converted into DSBs (33,34). A recent study using BRCA2 separation of function mutants proficient in HR but deficient in FP and RGS indicated that the ultimate determinant of chemosensitivity is HR-mediated DSB repair (17). Overall, these findings point to the model that accumulation of ssDNA gaps drives chemosensitivity in HR-deficient cells through their conversion into DSBs, which cannot be repaired in these cells.

Our findings broaden the understanding of histone chaperone-dependent resistance mechanisms. They also broaden the patient population potentially exhibiting this resistance mechanism from only patients with the limited genetic background allowing for the HIRA-dependent compensatory nucleosome establishment mechanism to any patient harboring a BRCA-deficient cancer with genetic background resulting in the observed PrimPol recruitment defect. Altogether, our work demonstrates that the CAF1–ASF1A pathway is key to promoting ssDNA gap accumulation, implying that knowing the status of CAF-1 and ASF1A expression within HR-deficient tumors is clinically relevant and will be advantageous for prediction of response to genotoxic therapies such as cisplatin.

## Supplementary Material

gkae1068_Supplemental_Files

## Data Availability

The source data underlying all figures and Supplementary Figures are provided in Supplementary Table S1.
