# Supplementary material for: CAF-1 promotes efficient PrimPol recruitment to nascent DNA for single-stranded DNA gap formation
Source: Nucleic Acids Res. 2024 Nov 18;52(22):13865–80. doi: 10.1093/nar/gkae1068 (PMC11662685; doi:10.1093/nar/gkae1068)
Supplement: gkae1068_Supplemental_Files [file gkae1068_supplemental_files.zip › Supplementary Material.pdf]

SUPPLEMENTARY MATERIAL

Legends to Supplementary Tables

Supplementary Table S1. The source data underlying each of the main and supplementary figure panels, including: the values plotted in graphs, the exact p-values, and the uncropped blots.

Supplementary Table S2. MAGeCK analyses of the CRISPR screen identifying genes whose loss decreases survival of CHAF1A^{KO} 293T cells.

Supplementary Table S3. MAGeCK analyses of the CRISPR screens identifying genes whose loss increases cisplatin sensitivity in 293T cells.

Supplementary Table S4. MAGeCK analyses of the CRISPR screens identifying genes whose loss increases the survival of CHAF1A^{KO} 293T cells in the presence of cisplatin.

Supplementary Figure S1

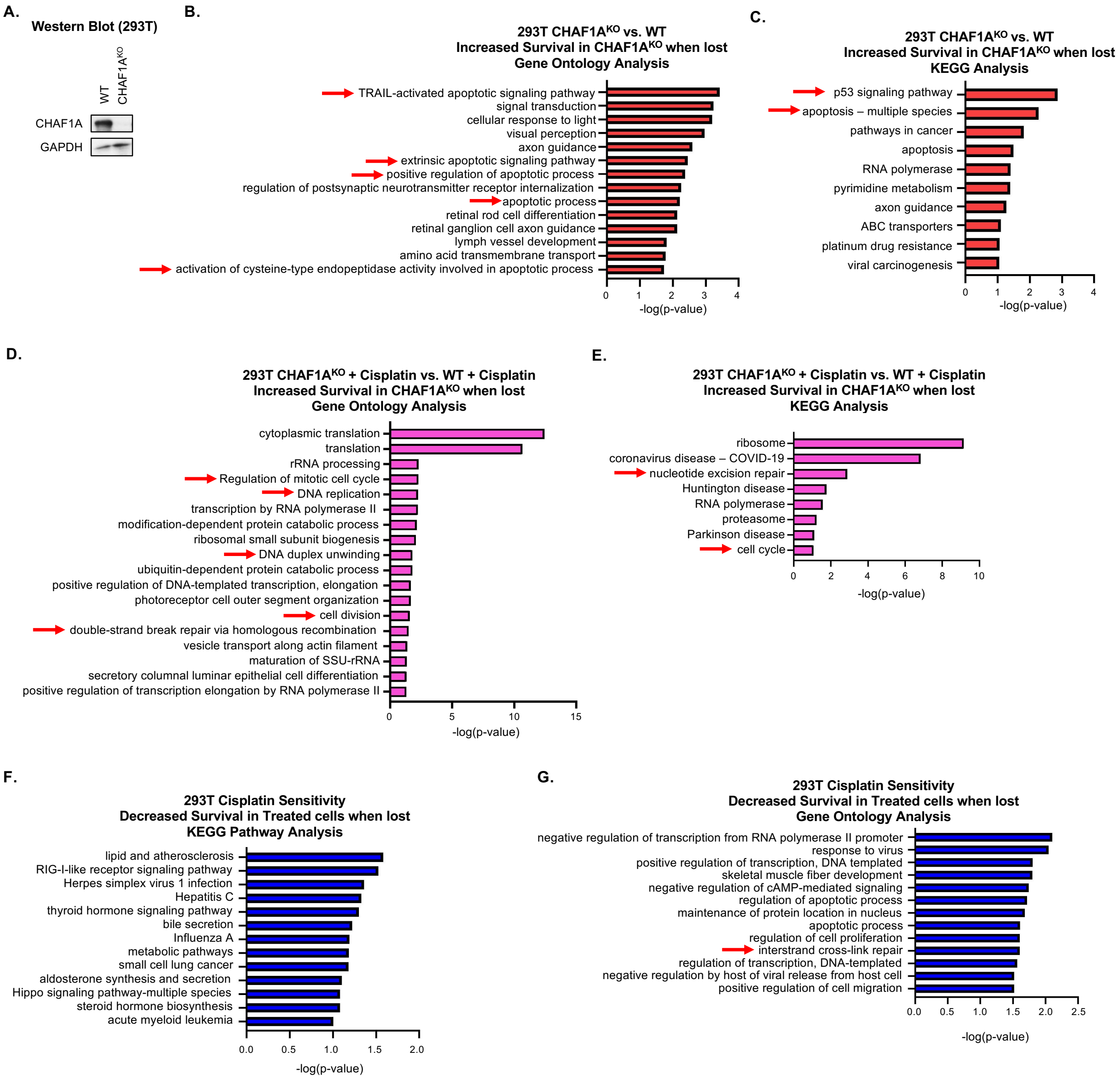

Supplementary Figure S1. Results of cisplatin sensitivity screens.

A. Western blot showing CHAF1A knockout in 293T cells.

B,C. Overrepresentation analysis of biological pathways of the top hits with p<0.02 that when lost result in increased survival of CHAF1A^{KO} 293T cells. Gene Ontology **(B)** and **(C)** KEGG terms with negative logP greater than 1.70 and 1.00 respectively are presented.

D,E. Overrepresentation analysis of biological pathways of the top hits with $p<0.02$ that when lost increase survival of CHAF1A^{KO} 293T cells compared to WT 293T cells when in the presence of 0.625 μM cisplatin. Gene Ontology **(D)** and **(E)** KEGG terms with negative logP greater than 1.30 and 1.00 are presented.

F,G. Overrepresentation analyses of biological pathways of the top hits with $p<0.02$ that result in sensitivity to cisplatin in 293T WT cells. KEGG **(F)** and Gene Ontology **(G)** terms with negative logP greater than 1.00 and 1.50 respectively are presented.

Supplementary Figure S2

A.

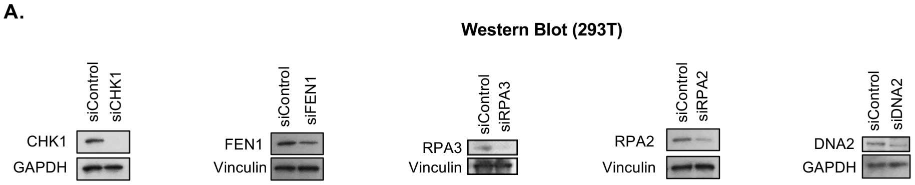

Supplementary Figure S2. Confirmation of Top Hit gene knockdowns.

A. Western blots showing depletion of selected top hits CHK1, FEN1, RPA3, RPA2, DNA2 in WT 293T cells.

Supplementary Figure S3

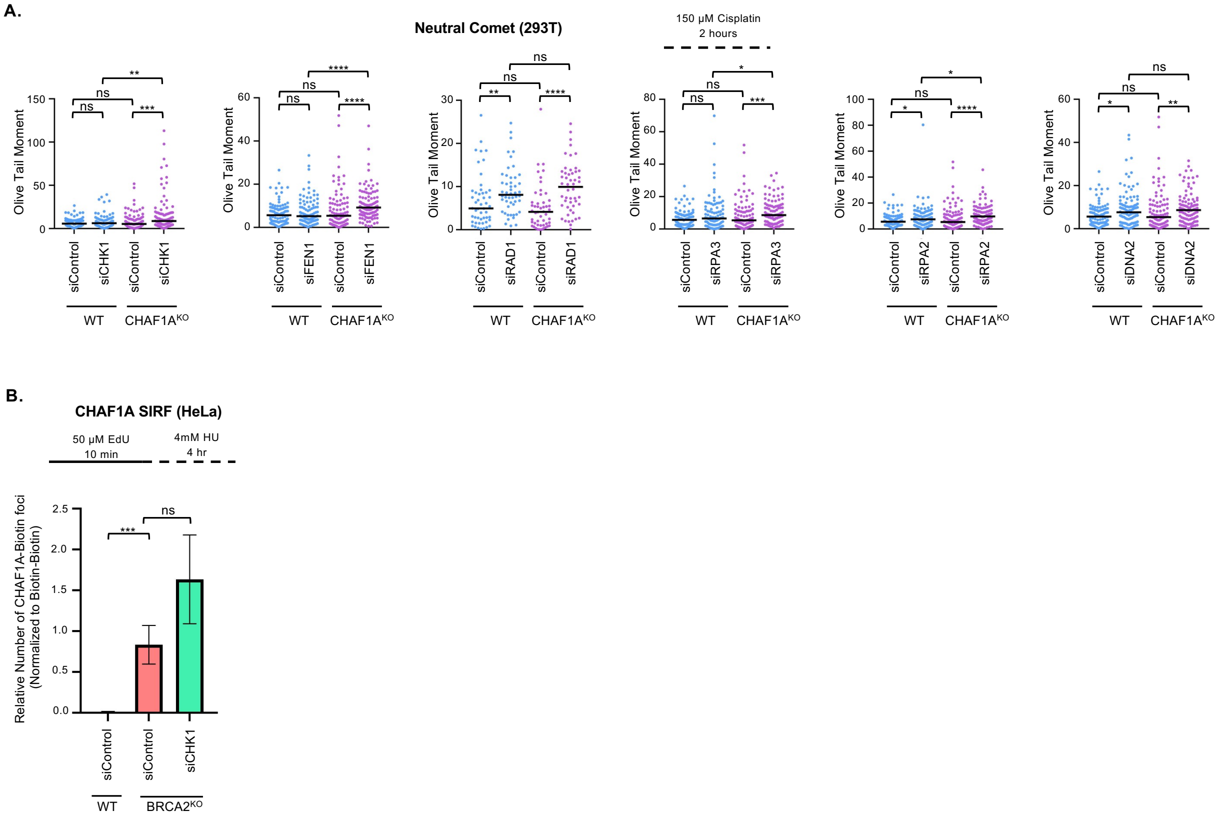

Supplementary Figure S3. Additional functional investigation of Top Hits from CRISPR screen.

A. Neutral comet assay showing that depletion of top hits results in a larger increase in DSB formation in CHAF1A^{KO} cells than in wildtype 293T cells. At least 50 nuclei were quantified per condition with median values marked on the graph. Asterisks indicate statistical significance (Mann-Whitney, two-tailed). A schematic representation of the assay conditions is displayed.

B. SIRF assay showing that sequestration of CHAF1A behind the replication fork under fork stalling conditions previously reported in BRCA2^{KO} HeLa cells is further increased following the depletion of CHK1. At least 100 cells were quantified for each condition. Bars indicate the mean values, error bars represent SEM, and asterisks indicate statistical significance (t-test, two-tailed, unpaired). A schematic representation of the assay conditions is shown.

Supplementary Figure S4

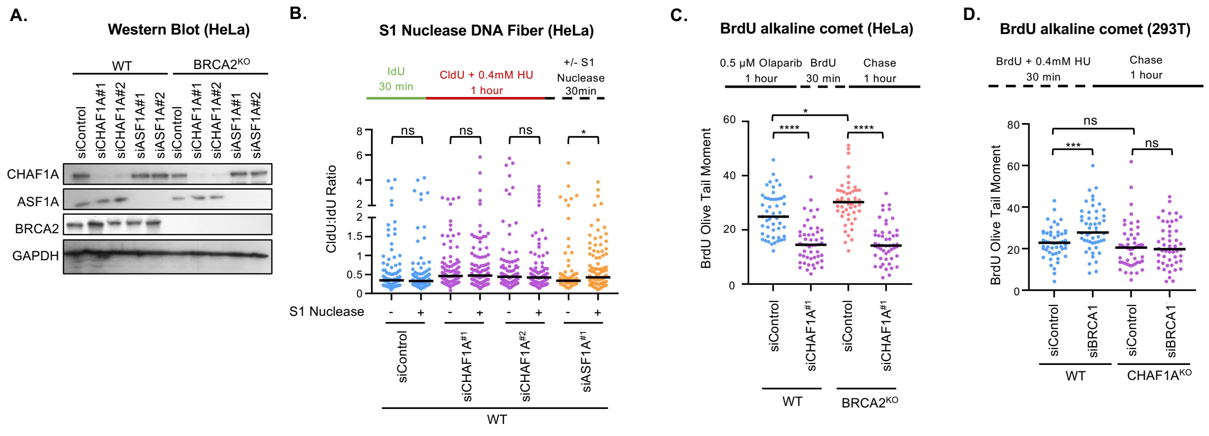

Supplementary Figure S4. Impact of CHAF1A on gap formation.

A. Western blot showing depletion of CHAF1A and ASF1A in both WT and BRCA2^{KO} HeLa cells.

B. S1 Nuclease DNA fiber combing assay showing that depletion of CHAF1A or ASF1A in WT HeLa cells increases the CldU:IdU ratio similarly to BRCA2^{KO} HeLa cells after treatment with 0.4 mM HU. At least 99 fibers were quantified per condition. The ratio of CldU to IdU tract lengths is presented with median values marked. Asterisks indicate statistical significance (Mann Whitney test, two-tailed). A schematic representation of the assay conditions is displayed.

C. BrdU alkaline comet showing that depletion of CHAF1A in WT or BRCA2^{KO} HeLa cells results in a decrease in ssDNA gap accumulation after 1 hour pretreatment with 0.5 μM Olaparib. At least 49 nuclei were quantified per condition with median values marked on the graph. Asterisks indicate statistical significance (Mann-Whitney, two-tailed). A schematic representation of the assay conditions is shown.

D. BrdU alkaline comet showing that depletion of BRCA1 in CHAF1A^{KO} 293T cells does not result in an increase in ssDNA gap accumulation as it does in WT 293T cells upon treatment

with 0.4 mM HU. At least 50 nuclei were quantified per condition with median values marked on the graph. Asterisks indicate statistical significance (Mann-Whitney, two-tailed). Schematic representations of the assay conditions are displayed.

Supplementary Figure S5

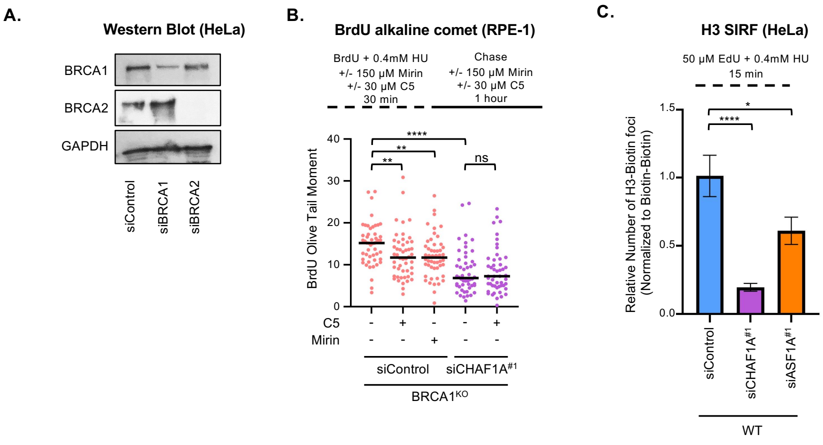

Supplementary Figure S5. Impact of CHAF1A on histone H3 deposition.

A. Western blot showing knockdown of BRCA1 and BRCA2 in HeLa cells.

B. BrdU alkaline comet assay showing that inhibition of DNA2 via 30 μM C5 treatment is epistatic with the decrease in ssDNA gap accumulation observed with knockdown of CHAF1A or ASF1A in BRCA1^{KO} RPE1 cells. At least 50 nuclei were quantified per condition with median values marked on the graph. Asterisks indicate statistical significance (Mann-Whitney, two-tailed). Cells were incubated with or without mirin or C5 during the BrdU incubation period as well as with their respective mirin conditions during the following 1-hour chase as shown in the schematic representation of the assay conditions displayed.

C. SIRF assay showing that localization of H3 to nascent DNA in HeLa cells decreases when either CHAF1A or ASF1A are knocked down. At least 100 cells were quantified for each condition. Bars indicate the mean values, error bars represent SEM, and asterisks indicate statistical significance (t-test, two-tailed, unpaired). A schematic representation of the assay conditions is shown.

Supplementary Figure S6

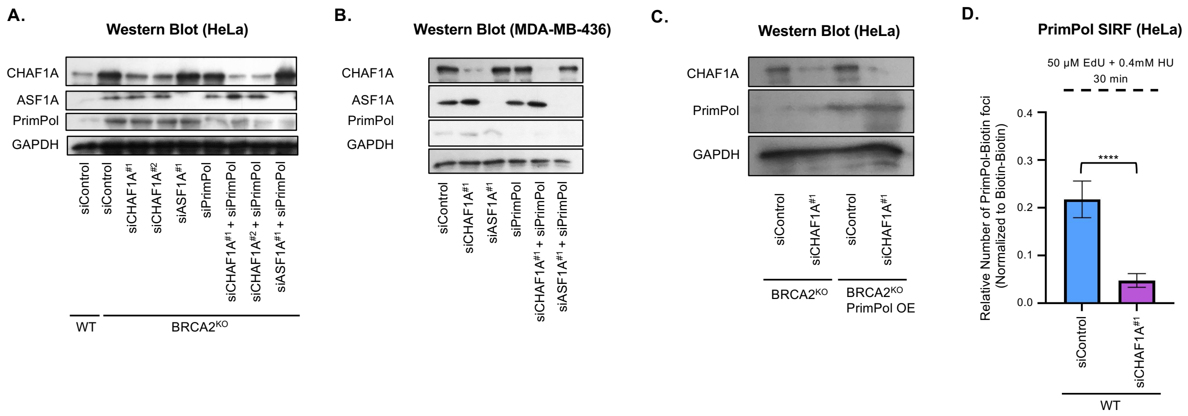

Supplementary Figure S6. Confirmation of gene knockdowns.

A. Western blot showing co-depletion of either CHAF1A or ASF1A and PrimPol in both WT and $BRCA2^{KO}$ HeLa cells.

B. Western blot showing co-depletion of either CHAF1A or ASF1A and PrimPol in MDA-MB-436 cells.

C. Western blot showing depletion of CHAF1A in both HeLa $BRCA2^{KO}$ and $BRCA2^{KO}$ PrimPol overexpressing cells.

D. SIRF assay showing that PrimPol localization to nascent DNA upon 0.4 mM HU treatment is decreased in WT HeLa cells similarly to BRCA2KO cells when CHAF1A is depleted. At least 100 cells were quantified for each condition. Bars indicate the mean values, error bars represent SEM, and asterisks indicate statistical significance (t-test, two-tailed, unpaired). A schematic representation of the assay conditions is shown.
